# Involuntary Autobiographical Memory Chains: Implications for Autobiographical Memory Organization

**DOI:** 10.3389/fpsyt.2014.00183

**Published:** 2014-12-16

**Authors:** John H. Mace

**Affiliations:** ^1^Department of Psychology, Eastern Illinois University, Charleston, IL, USA

**Keywords:** involuntary memory chains, autobiographical memory, autobiographical memory organization, involuntary memory, episodic memories

Sometimes when we unintentionally or intentionally retrieve a memory of a past episode, we experience one or more additional memories, which spring to mind quickly and uncontrollably. For example, one might involuntarily remember seeing mummies in the British Museum, and this memory could in turn trigger a memory of seeing the Egyptian collection at the natural history museum in New York City. Known as an involuntary autobiographical memory chaining (1, 2), this memory phenomenon is probably common in everyday life when we are engaged in controlled, voluntary recall of the past, and when memories just come to mind unintentionally [Ref. (1–3); see also (4–6), for reviews of involuntary remembering]. Involuntary memory chaining has not been researched as much as singly experienced involuntary memories (i.e., the case where an involuntary memory does not result in additional involuntary retrievals), so comparatively little is known about them. However, studies that have focused on involuntary memory chains have turned up findings with interesting implications for theories of autobiographical memory organization (7, 8). In this article, I review how these findings have helped develop a theory of autobiographical memory organization that posits that episodic memories are organized as conceptual classes of events.

I have argued that involuntary memory chains are the products of spreading activation in the autobiographical memory system (2, 6). Thus, when a memory is activated in the autobiographical memory system (e.g., as a result of voluntary or involuntary retrieval), this activation spreads to other memories in its associative network. Normally, these types of activations do not come into consciousness because they are either too weak or irrelevant to one’s current cognitive activity. However, when activations are strong enough, or relevant, they come into consciousness where they are experienced as chained involuntary memories.

Although we have no firm evidence that involuntary memory chains are spreading activations, there are a number of reasons why we should see this as a good, tentative explanation. For one, there is good reason to believe that like semantic memories, memories in the autobiographical memory system are networked (or connected) and therefore capable of activating one another. Thus, like semantic memory, once memories are activated in autobiographical memory, there is an obligatory spread of activations to neighboring, related memories within a network. Evidence that such an architecture exists in autobiographical memory can be found in priming studies [e.g., Ref. (3, 9, 10)]. For example, Mace (3) and Mace and Clevinger (8) have shown that activating autobiographical memories in voluntary recall task primes the subsequent recall of related memories at some future point.

Additionally, there is good reason to doubt that involuntary memory chains are driven by some sort of alternative retrieval process other than spreading activation. For example, the most obvious alternative explanation is that a single retrieval cue simply triggers more than one memory, and since both memories cannot come to mind at once, they are experienced in sequence as a chain of memories. If this view were true, then memories in involuntary memories chains should almost always be related to the retrieval cue that trigger the first memory, however, just the opposite is true [see Discussion in Ref. (2)].

Viewing involuntary memory chains as an automatic retrieval process akin to spreading activation has led us to assert that the products of involuntary memory chains (i.e., the memories found in a chain) are reflective of the underlying organization of autobiographical memory (7, 8). Thus, examining their output should prove elucidating to the study of autobiographical memory organization.

To date, the output of involuntary memory chains has been observed in two types of settings, in the laboratory, where participants reported involuntary memory chains, resulting from voluntary retrievals, and in naturalistic settings, where participants reported involuntary memory chains, resulting from involuntary retrievals (i.e., involuntary memories experienced in everyday life). In both laboratory and naturalistic measures, involuntary memory chains have consistently exhibited two types of associative forms: general-event associated memories or conceptually associated memories.

In general-event associations, memories in a chain come from the same general (or extended) event period [i.e., a general memory such as the night at the opera, a trip to London; or a summary memory, repeated events such as Sunday walks in the park, see Ref. (11, 12)]. These memories appear to be connected by the larger general or summary event, and therefore, are typically temporally proximate (e.g., spanning the same day, evening, week, month, etc.). In contrast, conceptual associations are associated by their overlapping content. For example, they commonly involve the same people, objects, activities, or other common themes (such as work or school). Conceptually associated memories can span any time period, and therefore, temporality does not appear to be their main organizing principle.

Analyses of the relative proportions of conceptually associated and general-event associated involuntary memory chains have highlighted the importance and dominance of conceptually associations. In both laboratory and naturalistic measures, conceptually associated memories have significantly outnumbered general-event associated memories, with conceptual associations usually exceeding 80% (1, 2, 7, 8). Other analyses and measures have also revealed conceptual association dominance [see Ref. (7, 8)], and while these analyses alone could make a strong case for the idea that episodic memories are contained mostly in conceptual associative networks, retention interval analyses of conceptually and general-event associated memories have made a more cogent case.

In Mace et al. (8), involuntary memory chains were divided into three retention intervals, chains with memories up to a year old, 1–9 years old, and over 9 years. Analyses of conceptually and general-event associations showed the two categories were equal when memories were up to a year old (53% conceptual, 47% general event), but their relative proportions changed dramatically when memories were over a year old (1–9 years, 77%, conceptual, 23%, general event; over 9 years, 85%, conceptual, 15%, general event). These data make a stronger case for a basic conceptual organization than the simpler data presented above because they show that conceptual associations do not just simply outnumber general-event associations because there are more of the former type of experiences, but because the basic architecture fundamentally favors forming and maintaining conceptual consolidations.

For example, let us consider this proposition with memories of being at a wedding. When one remembers an episode from a wedding they had recently attended (e.g., within the last few weeks or months), this memory has a 50% chance of triggering others episodes from that same event, and a 50% chance of triggering memories of having attended other weddings. However, when one recalls that same episode some time later (e.g., 2 years later), it now has an 80% chance of triggering memories of other weddings. According to the conceptual organization account, this happens because memories of the wedding initially consolidate together (as ageneral-event network of the wedding), as well as with memories of other weddings (joining the existing conceptual network for wedding memories). However, as time passes, some or all of the episodes from that memory are forgotten, leaving the connections to other wedding memories. Thus, events always consolidate into their conceptual class (e.g., all wedding memories), and it is these connections (or associations) that endure over time, not the temporal ones (i.e., memories from a particular wedding). In this account, general-event memories are lost quickly because they have a limited, short-term relevance. So, in the short-term it may be equally important to remember episodes from a wedding, or those of other weddings, while in the long term, it is more important to remember that one has been to various weddings.

In sum, then, these various finings showing the dominance of conceptual associations have led us to assert that autobiographical memories are organized primarily, or fundamentally, along conceptual lines. While temporal associations are clearly a part of autobiographical memories organization [see Ref. (3, 8)], they are not retained over time as conceptual associations are. It is important to emphasize that we are not claiming that autobiographical memories are a collection of abstract or categories association as in semantic memory. Instead, our use of the term conceptual refers to, for lack of a better term, experiential type concepts, such as people, places, locations, activities, and so forth. Thus, events coalescence according to their many and varied experiential similarities [see data in Ref. (8)].

In closing, the study of involuntary memory chains has been informative to the study of autobiographical memory organization. While I have presented some interpretations of the findings, clearly there are more, and more work is certainly needed to fully flesh them out. I have also argued (here and elsewhere) that involuntary memory chains are spreading activations that are normally unconscious, but for some reason they become conscious. This idea is interesting in its own right, and research that could elucidate why such activations sometimes surface into consciousness is likely to be informative to understanding involuntary retrievals and other areas of cognitive science and mental experience.

## Conflict of Interest Statement

The author declares that the research was conducted in the absence of any commercial or financial relationships that could be construed as a potential conflict of interest.
